# Dual Delivery of TGF-β3 and Ghrelin in Microsphere/Hydrogel Systems for Cartilage Regeneration

**DOI:** 10.3390/molecules26195732

**Published:** 2021-09-22

**Authors:** Jianjing Lin, Li Wang, Jianhao Lin, Qiang Liu

**Affiliations:** 1 Arthritis Clinical and Research Center, Peking University People’s Hospital, No. 11 Xizhimen South Street, Beijing 100044, China; linjianjing@bjmu.edu.cn (J.L.); linjianhao@pkuph.edu.cn (J.L.); 2 Arthritis Institute, Peking University, Beijing 100044, China; 3 Department of Biomedical Engineering, College of Engineering, Peking University, Beijing 100871, China; wanglious@pku.edu.cn

**Keywords:** dual delivery, TGF-β3, ghrelin, microspheres, hydrogel, cartilage regeneration

## Abstract

Articular cartilage (AC) damage is quite common, but due to AC’s poor self-healing ability, the damage can easily develop into osteoarthritis (OA). To solve this problem, we developed a microsphere/hydrogel system that provides two growth factors that promote cartilage repair: transforming growth factor-β3 (TGF-β3) to enhance cartilage tissue formation and ghrelin synergy TGF-β to significantly enhance the chondrogenic differentiation. The hydrogel and microspheres were characterized in vitro, and the biocompatibility of the system was verified. Double emulsion solvent extraction technology (*w*/*o*/*w*) is used to encapsulate TGF-β3 and ghrelin into microspheres, and these microspheres are encapsulated in a hydrogel to continuously release TGF-β3 and ghrelin. According to the chondrogenic differentiation ability of mesenchymal stem cells (MSCs) in vitro, the concentrations of the two growth factors were optimized to promote cartilage regeneration.

## 1. Introduction

Articular cartilage (AC) injury is very common, and the incidence of AC injury was found to be 57% in 1000 patients undergoing knee arthroscopy [1]. As a tissue without blood supply, nerve tissue or lymph nodes, ACs lack a self-healing ability [2]. Once an AC is injured, without proper and timely intervention, AC damage may develop into osteoarthritis (OA). The treatment of cartilage injury has been extensively studied since the last century [3], such as bone marrow-stimulating techniques, osteochondral transplantation, autologous chondrocyte implantation (ACI) and stem cells transplantation, however, these methods have disadvantages such as a lack of biomechanical and viscoelastic characteristics of the cartilage produced, limited donor sources, complicated surgery, long recovery time and potential tissue malformations in vivo [4]. Therefore, no treatment has been established as the gold standard. Therefore, there is an urgent need to develop new treatments for AC injury.

As the main components of tissue engineering, scaffolds and growth factors are promising methods for cartilage regeneration. Due to its high viscoelasticity similar to natural tissues, in situ gelling property and biodegradability, hydrogels are attractive materials for cartilage regeneration [5]. Growth factors provide an appropriate microenvironment for the growth of cartilage tissue. They can promote the regeneration of articular cartilage by enhancing the recruitment and differentiation of mesenchymal stem cells (MSCs) [6]. Transforming growth factors beta, also known as TGF-βs, are key components of the chondrogenic differentiation of stem cells and subsequent cartilage regeneration [7,8]. Among them, TGF-β3 is most often used in combination with delivery systems to inhibit the hypertrophy and apoptosis of chondrocytes. However, direct injection of TGF-β3 into the joints produces certain side effects, such as causing osteophyte formation [9]. Therefore, a combination of multiple growth factors have been explored to effectively promote chondrogenic differentiation and cartilage regeneration in cartilage in vivo [10] and in vitro [11]. Ghrelin is a polypeptide hormone mainly derived from the stomach and is the first endogenous ligand of growth hormone secretagogue receptor-1a (GHSR-1A) discovered so far [12]. We just found that ghrelin significantly enhances chondrogenic differentiation both in vitro and in vivo in conjunction with TGF-β3 [13]. Although hydrogels can deliver growth factors, the hydrophilicity of hydrogels makes the release of hydrophilic molecules too fast. Therefore, hydrogels have limited applications in delivering growth factors over a long period of time [5]. Therefore, the strategy for delivering these molecules is usually physical encapsulation and absorption. In order to alleviate burst release, microspheres containing these two molecules can partly overcome this problem [14].

In this study, a dual delivery system for TGF-β3 and ghrelin was prepared to achieve the co-delivery of therapeutic growth factors. Double-emulsion solvent extraction technology (*w*/*o*/*w*) was used to encapsulate TGF-β3 and ghrelin into microspheres, which were encapsulated in a methacrylated hyaluronic acid (HAMA) hydrogel. We studied the gelation, structural properties and in vitro release of the composite system. In addition, according to the chondrogenic differentiation ability of MSCs in vitro, the concentrations of the two growth factors were optimized.

## 2. Results

### 2.1. Study Design

**Figure 1:**
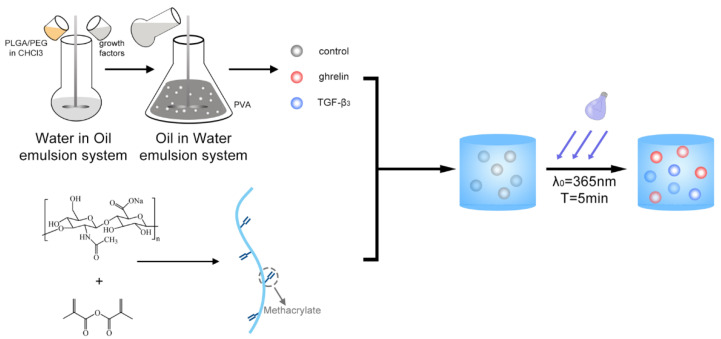
Schematic of the microsphere/hydrogel system.

The Poly (ethylene glycol) (PEG)/poly (lactic-co-glycolic acid) (PLGA) microspheres containing TGF-β3 or ghrelin were fabricated following the process shown in Figure 1.

### 2.2. Fabrication and Characterization of PEG/PLGA Microspheres

**Figure 2:**
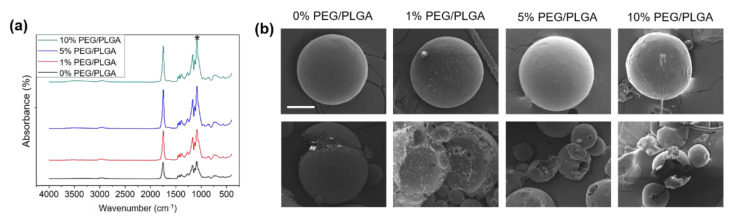
Fabrication and characterization of PEG/PLGA microspheres: (**a**) FTIR spectra of 0%, 1%, 5% and 10% PEG/PLGA microspheres. * represents the peak of the C–O–C bond from PEG; (**b**) SEM images of external and internal morphologies of the microspheres, where 0%, 1%, 5% and 10% are the mass ratios of the PEG/PLGA feed during preparation of microspheres. Scale bars: 100 μm. Abbreviations: PEG, poly (ethylene glycol); PLGA, poly (lactic-co-glycolic acid).

The peak of the C–O–C bond from PEG at 1160 cm^−1^ was increased with the increase in PEG content (Figure 2a), which suggested that the different PEG/PLGA microspheres were successfully obtained. The external and internal morphologies of the microspheres were obtained by SEM (Figure 2b). The image shows that the microspheres have a smooth surface and a porous structure inside.

### 2.3. Fabrication and Characterization of the Microsphere/Hydrogel System

**Figure 3:**
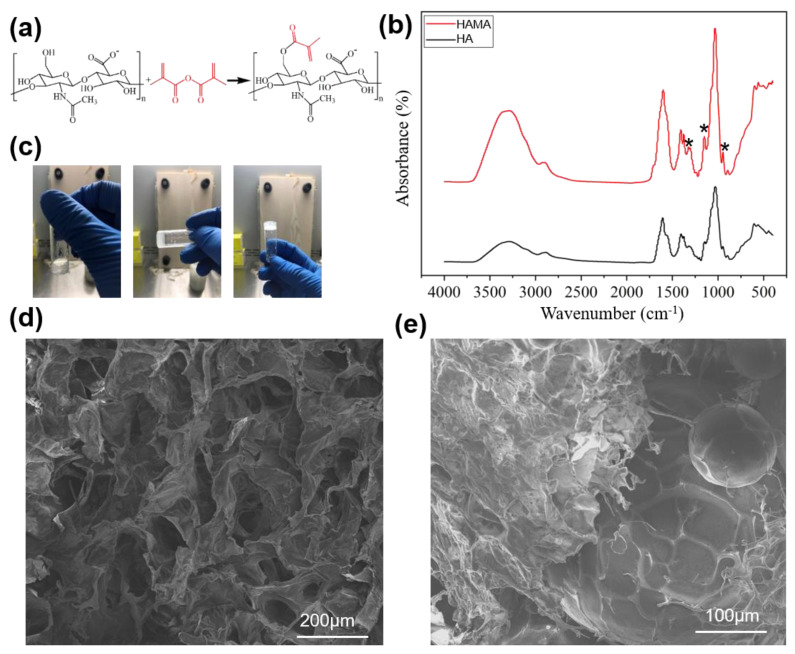
Fabrication and characterization of the microsphere/hydrogel system: (**a**) schematic illustration of the synthetic HAMA; (**b**) FTIR spectra of HA and HAMA hydrogels, where * represents the peak of the stretching vibrations of the C–O bond and C–O–C ether bond, and the bending vibration of the C=C bond; (**c**) gelation of the HAMA hydrogel; (**d**) SEM images of the internal structure of the HAMA hydrogel; and (**e**) SEM images of the microsphere/hydrogel system. Abbreviations: HA, hyaluronic acid; HAMA, methacrylated hyaluronic acid.

The HAMA hydrogel was fabricated as shown in Figure 3a. After methacrylation, the peak at 1376 cm^−1^ (bending vibration of C–H in CH3), 1323 cm^−1^ (stretching vibration of the C–O bond), 1148 cm^−1^ (C–O–C ether bond) and 946 cm^−1^ (bending vibration of the C=C bond) increased significantly, indicating that the C=C bond was successfully introduced (Figure 3b). Moreover, the peak of the stretching vibration of C=C bond at 1670~1600 cm^−1^ was low and was covered by the peak of the C=O bond of the amide bond, but the peak was still increased slightly, indicating C=C bond introduction and new C=O bond formation.

Under the action of a photoinitiator I2959, the HAMA formed a gel after 5 min of ultraviolet irradiation (Figure 3c). The morphology of the HAMA hydrogel showed a complete macroporous structure with good connectivity (Figure 3d). Mixed with microspheres, the HAMA hydrogel could wrap the microspheres and give the microspheres stable support (Figure 3e).

### 2.4. Biocompatibility Test of the Microsphere/Hydrogel System

**Figure 4:**
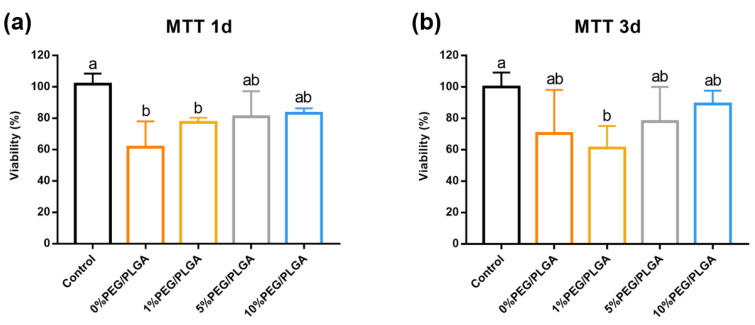
Biocompatibility test of the microsphere/hydrogel system. Cell viability of L929 cultured with day 1 (**a**) and day 3 (**b**), with the culture dish as the control, measured by an MTT assay. Statistical significance was marked with different letters (*p* < 0.05). Abbreviations: MTT, 3-(4,5-dimethylthiazol-2-yl)-2,5-diphenyl-2H-tetrazolium bromide.

The biocompatibility of the microsphere/hydrogel system was measured by MTT assay. Compared with a culture dish, the microspheres/hydrogel group showed 60–80% cell viability after 1 day of culture (Figure 4a). By day 3, the 10% PEG/PLGA showed the closest biocompatibility to the control group (Figure 4b). Regardless of the measurement being taken on day 1 or day 3, 10% PEG/PLGA showed the closest biocompatibility to the control group.

### 2.5. Study of the Controlled Release of TGF-β3 and Ghrelin in the Microsphere/Hydrogel System

**Figure 5:**
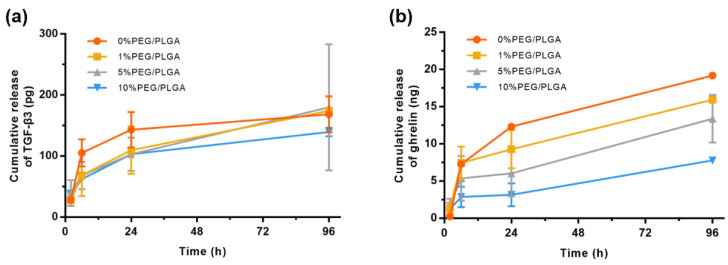
Study of the controlled release of TGF-β3 and ghrelin in the microsphere/hydrogel system in vitro. In vitro release of TGF-β3 (**a**) and ghrelin (**b**) from the microsphere/hydrogel system.

The microsphere hydrogel system achieved controlled release of TGF-β3 and ghrelin with burst release on the first day, followed by a stable release within 4 days (Figure 5). This pattern of release may be because the microspheres encapsulated on the surface of the hydrogel fell off, which led to the burst release on the first day. Then, the microspheres encapsulated inside the hydrogel were slowly released as the hydrogel degraded, so a stable and sustained release was achieved within 4 days. For the controlled release of TGF-β3, the microspheres with different PEG contents showed limited differences (Figure 5a). However, for ghrelin, the introduction of PEG significantly slowed the release rate of the drug (Figure 5b).

### 2.6. Identification of Human Bone Marrow Mesenchymal Stem Cells (hMSCs)

**Figure 6:**
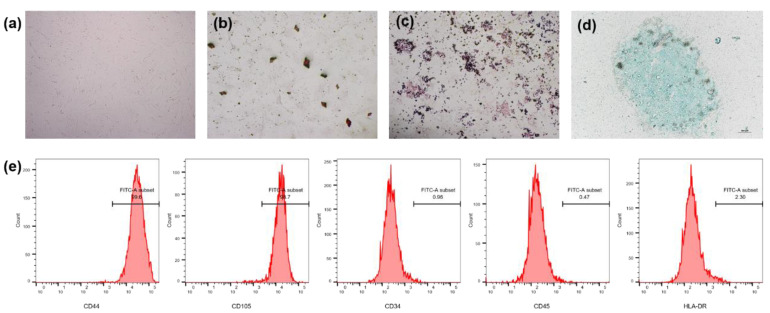
Identification of hMSCs. (**a**) Morphology of hMSCs in passage 5 under a microscope; (**b**) osteogenic differentiation of hMSCs stained with Alizarin Red; (**c**) adipogenic differentiation of hMSCs stained with Oil Red O; (**d**) chondrogenic differentiation of hMSCs stained with Alcian Blue; and (**e**) flow cytometry of MSC surface markers CD44 and CD105, and hematopoietic cell markers CD34, CD45 and HLA-DR of hMSCs in passage 5. Abbreviations: hMSCs, human bone marrow mesenchymal stem cells.

hMSCs showed a uniform spindle-shaped morphology, with a typical spiral arrangement under the microscope (Figure 6a). After 14 days of culture in the three-line differentiation induction medium, Alizarin Red S staining (Figure 6b), Oil Red O staining (Figure 6c) and Alcian Blue staining (Figure 6d) confirmed that hMSCs have multidirectional differentiation ability. The results of flow cytometry showed that hMSCs expressed MSC surface markers CD44 and CD105 but did not express hematopoietic cell markers CD34, CD45 and HLA-DR (Figure 6e).

### 2.7. Combination of Different Concentrations of TGF-β3 and Ghrelin on the Chondrogenic Differentiation of hMSCs

**Figure 7:**
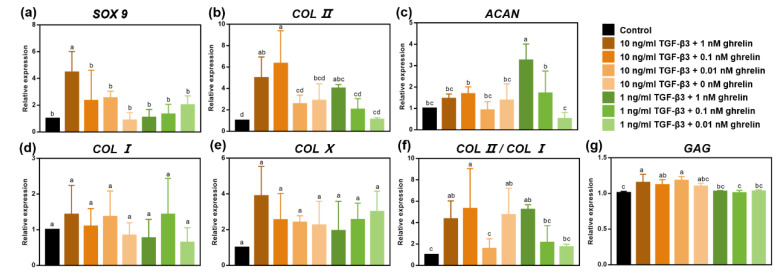
Effects of combinations of different concentrations of TGF-β3 and ghrelin on the chondrogenic differentiation of hMSCs. qRT-PCR analyses of SOX9 (**a**), COL II (**b**), ACAN (**c**), COL I (**d**), COL X (**e**), and COL II/COL I (**f**) expression and on the quantification of GAG (**g**) were conducted after 21 days of chondrogenic differentiation with different concentrations of TGF-β3 and ghrelin. Statistical significance was marked with different letters (*p* < 0.05). Abbreviations: SOX9, Sry-type high-mobility-group box 9; COL II, Type II collagen; ACAN, Aggrecan; COL I, Type I collagen; COL X, Type X collagen; GAG, glycosaminoglycan.

qRT-PCR results showed that the combination of 10 ng/mL TGF-β3 and 0.1 nM ghrelin, and the combination of 1 ng/mL TGF-β3 and 1 nM ghrelin significantly increased the expression of the chondrogenic differentiation gene Sry-type high-mobility-group box 9 (SOX9), Type II collagen (COL II) and Aggrecan (ACAN) (Figure 7a–c). They also reduced the expressions of hypertrophic indicators Type I collagen (COL I) and Type X collagen (COL X) and significantly increased the ratio of COL II/COL I (Figure 7d–f). The quantitative results of glycosaminoglycan (GAG) showed that the combination of 10 ng/mL TGF-β3 and 0.1 nM ghrelin significantly increased the expression of GAG (Figure 7g). In general, the combination of 10 ng/mL TGF-β3 and 0.1 nM ghrelin had a stronger chondrogenesis ability.

## 3. Discussion

As the three key components of tissue engineering, scaffolds, cells and signaling factors have gradually gained attention in the field of cartilage regeneration [15]. However, cartilage tissue engineering based on exogenous cells has many shortcomings that need to be solved, including its expensive cost, the cumbersome cell harvesting and expansion, potential immune rejection, and ethical issues [2,4]. In recent years, studies in vivo [10] and in vitro [11] have shown that a combination of multiple growth factors can be an effective inducer to promote cartilage damage repair. Unfortunately, rapid clearance of the joint cavity of the drug is still a key limitation affecting the efficacy of most therapeutic factors. This concept has been observed in various substances and animal species from small-molecule drugs to high-molecular-weight macromolecules [16]. Therefore, designing a growth factor delivery system to maintain growth factor levels in the joints for a long time is a concern. In this research, we developed a dual growth factor delivery system based on HAMA hydrogel and microspheres. The system used double-emulsion solvent extraction technology (*w*/*o*/*w*) to wrap TGF-β3 and ghrelin in microspheres and then mix the microspheres into the hydrogel. The results showed that the microsphere/hydrogel delivery system has good biocompatibility. With the help of this optimized delivery system, TGF-β3 and ghrelin are released slowly and in a controlled manner.

Biomimetic scaffolds to reconstruct the structural characteristics of natural articular cartilage are essential for successful cartilage regeneration. As a soluble polymer in a cartilage extracellular matrix (ECM) and joint synovial fluid, HA has become an excellent biomaterial for building a microenvironment that mimics the extracellular matrix of cartilage due to its good biocompatibility and bioactivity [17]. It is widely used for the treatment of cartilage regeneration and OA. HAMA gels are currently widely used in various tissue engineering applications including cartilage tissue engineering [18]. In our previous study, we used a HAMA hydrogel to induce chondrogenic differentiation of stem cells in vitro and significantly improved the cartilage regeneration ability in a rat osteochondral defect model. The cartilage defect was almost completely resolved in 4 weeks [19]. These findings indicate that we replicated the physiological structure of natural cartilage.

The HAMA hydrogel embedded with PEG/PLGA microspheres not only can mimic some of the characteristics of a native ECM but also can deliver bioactive molecules continuously and locally to realize long-term exposure of BMSCs to bioactive molecules during the cartilage repair process. Previous studies have shown that PLGA microspheres carrying TGF-β3 wrapped in an mPA hydrogel can continuously release TGF-β3 and can enhance the expression of chondrogenic genes [5]. In this study, we manufactured PLGA microspheres containing PEG at different concentrations. After culturing with L929 cells, we used the MTT test to find that the biocompatibility was good. After analyzing the controlled release of TGF-β3 and ghrelin, we used 10%PEG/PLGA microspheres for the follow-up experiments. Compared with simple PLGA microspheres, the controlled release of TGF-β3 was slower in 10%PEG/PLGA microspheres. For ghrelin, the introduction of 10%PEG significantly slowed down the drug release rate, which may be because PEG is more hydrophilic than PLGA and can better stabilize ghrelin, which is hydrophilic [20].

In our previous study, we studied the effects of the combination of 10 ng/mL TGF-β3 and different concentrations of ghrelin (1, 10 and 100 nM) on the chondrogenic differentiation of MSCs. The results indicated that ghrelin synergistically increased the chondrogenic effect of TGF-β3 in a negative dose-dependent manner. The lowest tested dose of 1 nM ghrelin significantly increased the gene expression levels of SOX9, COL II and ACAN. Ghrelin at other concentrations had a weaker synergistic effect on chondrogenic differentiation [13]. In this study, we continued to explore the effect of the combination of lower concentrations of ghrelin (1, 0.1 and 0.01 nM) and TGF-β3 (10 ng/mL and 1 ng/mL) on chondrogenic differentiation. Regarding the gene expression related to the chondrogenic differentiation of MSCs, our results proved that the combination of 10 ng/mL TGF-β3 and 0.1 nM ghrelin had the best effect on the chondrogenic differentiation of stem cells. In our future work, we will use the optimal dose of the TGF-β3 and ghrelin combination in the microsphere/hydrogel system to further verify the in vivo repair in an osteochondral defect model.

Our research has some limitations, which will be resolved in follow-up investigations. Our experiment only developed a dual delivery system of growth factors for cartilage regeneration and thus lacked verification of the animal experiments. Our previous cartilage repair experiment on the combination of TGF-β3, ghrelin and MSCs was performed in a rodent model using rats, which does not represent the repair in humans. The knee joints of adult mini-pigs are upright, their adult weight can grow up to 70 kg, and their physiological and repairing properties are similar to those of humans [10]. Therefore, before determining the clinical relevance of using the HAMA hydrogel embedded with PEG/PLGA microspheres in treating cartilage defects, it needs to be studied in larger animals, such as miniature pigs.

## 4. Materials and Methods

### 4.1. Materials

Poly (lactic-co-glycolic acid) (PLGA, Mw 24,000–38,000) was obtained from ShangDong Daigang BIO Engineer Limited. Poly (ethylene glycol) methyl ether (PEG) was obtained from Aldrich. Sodium hyaluronate (HA, Mw 100,000–200,000) and methacrylic anhydride were obtained from Heowns. Ghrelin (Phoenix Pharmaceuticals, 031-30), TGF-β3 (PeproTech, 100-21-10) and other regents were commercially available and used as received.

### 4.2. Fabrication of PEG/PLGA Microspheres

The PEG/PLGA microspheres were fabricated using a double emulsion solvent extraction method as previously described [21]. In brief, 1.55 g of PLGA was dissolved in 20 mL of dichloromethane (DCM). Additionally, 0 mg, 15.5 mg, 77.5 mg and 155 mg PEG were subsequently dissolved to acquire 0%, 1%, 5% and 10% PEG/PLGA microspheres, respectively. Then, 80 uL of a solution containing water, ghrelin or TGF-β3 was added to the 6 mL DCM solution and stirred by a high-speed homogenizer (20,000 rpm, 2 min). Then, the mixed emulsion was added to 500 mL of the 0.1% (*w*/*v*) PVA solution and agitated at a speed of 400 rpm for 5 min. Then, the resulting solution was poured into 500 mL of the 2% isopropanol solution and agitated at a speed of 150 rpm for 3 h to acquire the microspheres. The microspheres were washed twice using deionized water. After that, the microspheres were centrifuged and freeze-dried. The microspheres containing water, ghrelin or TGF-β were denoted as B-microspheres, G-microspheres and T-microspheres, respectively.

### 4.3. Fabrication of the HAMA Hydrogel and Microsphere/Hydrogel System

HAMA was synthesized as previous reported [22]. The HA was modified with a double bond by reacting with methacrylate. In brief, 1 g HA was dissolved into 100 mL water and agitated at a speed of 800 rpm until the HA was completely dissolved. Then, 1 mL of methacrylate was added, and the reaction was maintained for 12 h with the pH at 8~8.5. The whole process, including dissolution and reaction, was reacted on ice. After the reaction, we performed a 2D and freeze-dried dialyses on the solution. The microsphere/hydrogel system was formed by adding an appropriate amount of microspheres into 50 uL of the HAMA solution (2%, *w*/*v*, in PBS solution, 0.1% I2959) and then by exposure to 365 nm ultraviolet (UV) light (10 mW/cm^2^, 5 min).

### 4.4. Characterization of the Microsphere/Hydrogel System

The morphology of the microspheres and hydrogel were analyzed by scanning electron microscopy (SEM, Nova_NanoSEM430). The freeze-dried microspheres and hydrogel samples were mounted and coated with gold-palladium before being examined with SEM at an accelerating voltage of 5 kV. Characterization of the microspheres and HAMA hydrogel was performed with a Fourier-transform infrared spectrometer (FTIR, Nicolet is 50, Thermo Fisher, Waltham, MA, USA).

### 4.5. Study of TGF-β3 and Ghrelin Release from the Microsphere/Hydrogel System

The TGF-β3 and ghrelin loading capacities in the microspheres were determined using a solvent-extraction technique [23]. The concentrations of the extracted TGF-β3 and ghrelin were analyzed by enzyme-linked immunosorbent assay (ELISA) using a TGF-β3 Enzyme-linked Immunosorbent Assay Kit (Cloud-Clone, SEB949Hu-96T) and a Ghrelin EIA Kit (Phoenix EK-031-30). The loading capacity was calculated according to the following formula:

(1) $$\mathrm{Loading}\;\mathrm{capacity}=\frac{C_{e}V_{e}}{M_{e}},$$

where *M_e_* is the mass of the microspheres used for the ELISA tests, *V_e_* is the volume of solvent used in the extraction process, and *C_e_* is the drug concentration as measured by ELISA.

(2) $$\mathrm{Loading}\;\mathrm{efficiency}=\frac{C_{e}V_{e}M_{f}}{M_{e}M_{a}},$$

where *M_f_* is the mass of fabricated microspheres and *M_a_* is the mass of the drug added.

To analyze the TGF-β3 and ghrelin release dynamics of the microsphere/hydrogel system, appropriate amounts of T-microspheres or G-microspheres were added into 50 uL of the hydrogel and mixed. After exposure to 365 nm ultraviolet (UV) light (10 mW/cm^2^, 5 min), the microsphere/hydrogel system was washed with PBS solution three times and then incubated at 37 °C. The controlled release of TGF-β3 and ghrelin was detected at 2 h, 6 h, 24 h and 96 h via ELISA. The total amounts of TGF-β3 and ghrelin were calculated and converted according to the drug loading measured before.

### 4.6. Biocompatibility of the Microsphere/Hydrogel System

The biocompatibility of the microspheres/hydrogel system was measured using the MTT assay. After 1 day or 3 days of co-cultivation of the microsphere/hydrogel and fibroblast cell line L929, the MTT (3-(4,5-dimethylthiazol-2-yl)-2,5-diphenyl-2H-tetrazolium bromide, Sigma, St. Louis, MO, USA, 2128) solution was added and incubated at 37 °C for 3 h. Then, both the solution and the materials were removed, and each well was washed with PBS. DMSO (Invitrogen, Waltham, MA, USA) was added to lyse the cells, and the cell viability was calculated from the absorbance at 570 nm.

### 4.7. Cell Isolation and Culture

Human bone marrow mesenchymal stem cells (hMSCs) were obtained from OA patients undergoing total knee arthroplasty. All the procedures were approved with patient informed consent and approved by the Ethical Review Committee of Peking University People’s Hospital (2018PHC061). The hMSCs and L929 cell lines were cultured in Dulbecco’s modified Eagle medium (DMEM, Hyclone, Logan, UT, USA) supplemented with 10% fetal bovine serum (FBS, Gibico, Amarillo, TX, USA) and 1% penicillin/streptomycin (PS, Amresco, Dallas, TX, USA). The medium was changed every three days at 37 °C and 5% CO_2_.

### 4.8. Identification of hMSCs

Five-passage BMSCs were detected using the BD FACSCelesta flow cytometer (Becton Dickinson, Franklin Lakes, NJ, USA) to identify the MSC surface markers CD44 and CD105, and the negative markers CD34, CD45 and HLA-DR.

An osteogenic induction medium and an adipogenic induction medium were used to induce osteogenic and adipogenic differentiation of hMSCs. After 14 days, according to our previous study, the hMSCs were stained with Alizarin Red and Oil Red O [24]. For cartilage differentiation, MSC granules (2.5 × 10^5^ cells) [25] were induced by a cartilage induction medium for 14 days. Then, the pellets were frozen and cut into 8 µm slices and stained with Alcian Blue.

### 4.9. Chondrogenic Differentiation of hMSCs with Different Concentrations of TGF-β3 and Ghrelin

The effects of different concentrations of TGF-β3 (10  ng/mL and 1  ng/mL) and ghrelin (1 nM, 0.1 nM and 0.01 nM) on MSC chondrogenic differentiation were assayed. hMSCs at passage 5 (5 × 105 cells per hydrogel) were cultured in a serum-free chondrogenic differentiation medium with different concentrations of TGF-β3 and ghrelin. The culture medium was changed every 3 days. The samples were collected on day 21 for subsequent analysis.

### 4.10. Gene Expression Analysis

The total RNA was homogenized in the TRIzol reagent; then, the total RNAs were extracted following the manufacturer’s protocol. After determining the concentration of RNAs with an ND-1000 spectrophotometer (Nanodrop Technologies, Wilmington, DE, USA), quantitative real-time PCR was performed in the PCR system (Pikoreal 96, Thermo Fisher) with Real Master Mix SYBR Green (FP202, Tiangen, Beijing, China) following the manufacturer’s instructions. The gene-specific primers are listed in Table 1. The relative expression of each gene was expressed as fold-changes with respect to glyceraldehyde-3-phosphate dehydrogenase (GAPDH).

### 4.11. Quantification of Glycosaminoglycan (GAG)

After culturing in a chondrogenic differentiation medium for 21 days, the GAG content was measured using the DMMB assay. In brief, the cell-hydrogel compounds were digested in 50 mg/mL proteinase K at 56 °C overnight and heated at 90 °C for 10 min. After filtration, the solution was conjugated with DMMB, agitated for 30 min, and then centrifuged at 12,000 rpm for 10 min. Then, the centrifugal sediment was dissolved in a decomplexation solution, and the absorbance was measured at 630 nm. The GAG content was calculated according to a standard curve based on known chondroitin sulfate (27042-10G-F, Sigma) concentrations.

### 4.12. Statistical Analysis

Statistical analysis was performed using Prism 7 (one-way ANOVA). The data were expressed as the mean ± standard deviation. Statistical significance was marked with different letters (*p* < 0.05).

## 5. Conclusions

In this study, PEG/PLGA microspheres encapsulated in a HAMA hydrogel was found to have good biocompatibility and sustained release of TGF-β3 and ghrelin. It can provide a perfect microenvironment for cartilage regeneration in the body. In addition, different concentrations of TGF-β3 and ghrelin were screened for the optimal combination of cartilage-formation gene expression. This synthetic composite hydrogel system is a suitable candidate for prolonged articular cartilage regeneration, but it should be further evaluated in larger animal cartilage-defect models.

## Figures and Tables

**Table 1:** Details of the primer sequences.

Primers	Primer Sequence
GAPDH	Forward: GGGCTGCTTTTAACTCTGGT
	Reverse: GCAGGTTTTTCTAGACGG
Aggrecan	Forward: CACTGTTACCGCCACTTCCC
	Reverse: ACCAGCGGAAGTCCCCTTCG
Sox 9	Forward: AGCGAACGCACATCAAGAC
	Reverse: CTGTAGGCGATCTGTTGGGG
COL I	Forward: GACATGCTCAGCTTTGTGGA
	Reverse: CTTTGTCCACGTGGTCCTCT
COL II	Forward: CAGGTCAAGATGGTC
	Reverse: TTCAGCACCTGTCTCACCA
COL X	Forward: AGCCAGGGTTGCCAGGACCA
	Reverse: TTTTCCCACTCCAGGAGGGC

## Data Availability

The data presented in this study are available on request from the corresponding author. The data are not publicly available due to data protection protocol among researchers.
